# Does the direct anterior learning curve persist after fellowship training? A single-center comparative cohort study

**DOI:** 10.1186/s42836-026-00446-y

**Published:** 2026-11-05

**Authors:** Tiffany A. Smith, Alioune Diane, William K. Crockatt, Alexander L. Neuwirth, H. John Cooper, Nana O. Sarpong, Carl L. Herndon

**Affiliations:** Department of Orthopedic Surgery, Columbia University Medical Center, New York, NY 10032 USA

**Keywords:** Total hip arthroplasty, Direct anterior approach, Learning curve, Fellowship training, Complications, Operative time

## Abstract

**Introduction:**

The direct anterior approach (DAA) for total hip arthroplasty (THA) has historically been associated with a learning curve affecting both operative efficiency and early complications. Whether contemporary adult-reconstruction fellowship training modifies that curve is not established. We compared 90-day complication rates and operative times between newly graduated fellowship-trained DAA surgeons and one experienced DAA surgeon at a single high-volume center.

**Methods:**

This retrospective cohort study included 600 primary THAs performed via a DAA from a single institution between November 2019 and February 2025 with a minimum of 90-day follow-up. Intra- and post-operative complications were compared between the following two groups: the first 100 cases of each of three junior fellowship-trained arthroplasty surgeons (“Junior”) and the most recent 300 cases of a senior fellowship-trained arthroplasty surgeon (“Senior”). The study was powered to detect a 5% absolute difference in overall complication rates. Patient-reported outcome measures (PROMs) were analyzed as exploratory secondary endpoints.

**Results:**

Operative complications were infrequent in both cohorts, with a numerically higher rate in the Junior group that did not reach statistical significance (Junior 27 [9.0%] vs Senior 20 [6.7%]; absolute difference + 2.3%, 95% CI − 2.0% to + 6.6%, *p* = 0.362). No subgroup comparison reached statistical significance: DVT (1.0 vs 0.7%, *p* = 1.000), PE (0.3 vs 0.3%, *p* = 1.000), hematoma (0.3 vs 1.0%, *p* = 0.624), dislocation (1.3 vs 0.3%, *p* = 0.373), periprosthetic fracture (1.7 vs 1.0%, *p* = 0.725), neurapraxia (1.3 vs 2.0%, *p* = 0.752), prosthetic joint infection (1.0 vs 0.3%, *p* = 0.624), or wound complications (3.3 vs 2.0%, *p* = 0.448). All subgroup analyses were exploratory and underpowered for individually rare events. Medical events were infrequent and similar between groups (Junior 13 [4.3%] vs Senior 17 [5.7%], *p* = 0.575). Combining operative and medical complications, 40 events (13.3%) occurred in the Junior cohort and 37 (12.3%) in the Senior cohort (*p* = 0.807). Operative time was significantly longer among Junior surgeons (92.3 vs 68.5 min, *p* < 0.001). In exploratory analyses limited by 90-day PROM follow-up of 35% (Junior) and 48% (Senior), 90-day SF-12 and WOMAC scores were similar among respondents; given the incomplete follow-up, these descriptive findings are best regarded as hypothesis-generating rather than conclusive about functional recovery.

**Conclusion:**

Within a single high-volume center using standardized ERAS pathways, three fellowship-trained surgeons performing their first 100 DAA THAs had significantly longer operative times than one experienced DAA surgeon, with no evidence of a large difference in 90-day complication rates. Because the study was powered only to detect a 5% absolute difference, it cannot exclude smaller or rare-event differences and does not establish equivalence. These data generate the hypothesis that contemporary fellowship training combined with protocolized perioperative care shifts the early DAA learning curve toward efficiency rather than safety; confirmation requires an adequately powered, multicenter study.

## Introduction

Total hip arthroplasty (THA) is among the most successful procedures in orthopedic surgery, offering durable pain relief and functional restoration for end-stage hip disease [1]. The direct anterior approach (DAA), utilizing an intermuscular and internervous plane, has gained significant popularity over the past decade due to reports of reduced soft-tissue disruption, lower dislocation rates, and accelerated early recovery [2–4]. This trend is reflected in a large AAHKS member survey (996 respondents), in which 56.2% reported performing the DAA, with adopters tending to be earlier in practice, consistent with an ongoing generational shift toward the approach [5]. Whether the approach itself materially alters patient-reported recovery remains contested; Corradi et al. examined this using bilateral staged THA, in which each patient serves as their own control [6].

Complications reported early in DAA adoption cluster around femoral preparation, soft-tissue handling, and component positioning. Intraoperative femoral fractures, typically calcar, greater trochanteric, and diaphyseal splits occurring during exposure, broaching, or stem insertion, are increased in early experience and decline with case volume [7–10]. Lateral femoral cutaneous nerve neurapraxia and associated meralgia paresthetica are likewise more frequent early in practice, attributed to the course of the nerve across the anterior field near the ASIS, where it is vulnerable to retractor pressure and soft-tissue tension [9–14]. Acetabular component malpositioning, predisposing to impingement, instability, leg-length discrepancy, and early wear [15, 16], and superficial wound complications, plausibly related to longer operative times and unoptimized soft-tissue protocols [8, 17], follow the same pattern. Collectively, composite reoperation and revision risk concentrates within the first 50–100 cases and is driven largely by fracture and malposition [7, 8, 10, 18].

Importantly, most prior investigations assessed experienced arthroplasty surgeons converting to the DAA mid-career, often without formal fellowship training in the approach. Emerging literature suggests that dedicated fellowship training may mitigate this historical learning curve, yet few studies have directly compared newly graduated fellowship-trained surgeons performing their first independent cases against an established DAA surgeon working within the same institutional environment. We therefore compared 90-day complication rates and operative efficiency between these groups, testing the null hypothesis of no difference in complication rates. We note at the outset that a study of this size can exclude only large between-group differences.

## Methods

### Data collection

This retrospective cohort study included 600 primary, elective DAA total hip arthroplasty cases performed at a single academic institution between November 2019 and February 2025. Group 1 (“Junior”) consisted of the first 100 consecutive primary DAA cases performed by each of three junior fellowship-trained arthroplasty surgeons, resulting in 300 hips. Group 2 (“Senior”) consisted of the most recent 300 consecutive DAA cases performed by a single senior fellowship-trained surgeon. All four surgeons perform the DAA as their standard approach for primary elective THA, and all cases represented consecutive, unselected all-comer patients who were considered candidates for elective total hip arthroplasty. Intraoperative fluoroscopy was applied in a standardized fashion in all 600 cases: imaging was obtained after acetabular component impaction and after trial and final femoral component insertion to assess component position, leg length, and offset. No case in either cohort was performed without fluoroscopic verification. Three surgeons performed cases off-table while one junior surgeon performed cases on-table using a standard fracture table. Implant selection was not standardized, and there was appreciable variation both between and within surgeons. Femoral stem and acetabular component choice reflected individual surgeon preference and intraoperative judgement rather than protocol assignment. Across the series, femoral components were tapered stems of contemporary design, the cementless versions being hydroxyapatite-coated titanium and available in collared and collarless configurations, and the cemented versions being polished tapered designs; acetabular components were uncemented modular press-fit shells. Both cementless and cemented femoral fixation, including hybrid constructs in which the femoral component was cemented and the acetabular component was not, were permitted at the discretion of the operating surgeon; no protocol criteria governed this choice, which was typically made intraoperatively on the basis of bone quality. Implant choice was not a study variable, no comparison between systems was undertaken, and the study was neither designed nor powered to support any inference about the performance of a particular device.

Importantly, all junior surgeons were fully integrated into the institution’s established enhanced recovery after surgery (ERAS) protocols from the outset of their independent practice, utilizing the same standardized perioperative pathways as the senior surgeon. These pathways included standardized multimodal analgesia, perioperative anticoagulation protocols, and early mobilization strategies, ensuring that junior surgeons operated within an optimized, protocol-driven perioperative environment rather than developing individualized practices independently. All elective, primary DAA THA procedures with at least 90 days of follow-up were included. THAs performed for fracture, any revision surgeries, and patients with incomplete 90-day follow-up data were excluded. All three junior surgeons completed North American arthroplasty fellowships emphasizing DAA instruction with over 100 DAA THA cases logged during their year-long fellowship. The senior surgeon is over ten years into practice and has routinely performed the DAA throughout their career.

### Outcome variables

The primary outcomes were intra-operative and post-operative complications occurring within 90 days of surgery, including dislocation, periprosthetic fracture, prosthetic joint infection, wound complications, and neurapraxia. Bursitis and tendonitis were not included as complications. Secondary outcomes included operative time, length of stay, and medical complications including deep venous thrombosis (DVT), pulmonary embolism (PE), anemia requiring transfusion, urinary retention, acute kidney injury, stroke, myocardial infarction, and death, as well as patient-reported outcome measures (PROMs). PROMs, including SF-12 Physical and Mental component scores and WOMAC pain, stiffness, and function subscales, were collected preoperatively and at 90 days postoperatively using routinely administered institutional questionnaires. Operative time was calculated from surgical time-out to the end of surgical closure.

Each complication category was assigned an explicit operational definition before counts were compiled, so that the same rule could be applied to both cohorts and so that the categories are reproducible in other series. Complications were tabulated at the event level: a patient experiencing more than one complication contributed to each applicable category. Throughout this manuscript, “complication events” denotes the total number of events and “patients with at least one complication” denotes the patient-level proportion. The two are reported separately and are not interchangeable. Patient-level proportions were used for the logistic regression model. Return to the operating room was defined as any unplanned return to the operating room within 90 days of, and attributable to, the index procedure, including revision arthroplasty, open reduction of dislocation or periprosthetic fracture, operative wound revision, and debridement with implant retention. It is reported separately rather than within the composite, since it is a consequence of the complications already tabulated and counting it in the composite would double-count the patients concerned. Death is reported among the medical complications.

Hematoma was defined as a postoperative collection of blood documented clinically or radiographically. Operational definitions for the remaining complication categories are given below. Dislocation was defined as a radiographically confirmed complete loss of articulation between the femoral head and acetabular component within 90 days of surgery, whether treated by closed or open reduction. Periprosthetic fracture was defined as any fracture of the femur or acetabulum in contact with or immediately adjacent to a prosthetic component, identified intraoperatively or on imaging within 90 days and whether managed operatively or non-operatively; calcar fractures were included in this category, as were greater trochanteric and diaphyseal fractures. Prosthetic joint infection was defined as any deep infection requiring irrigation and debridement; superficial infection was captured separately under wound complications.

Neurapraxia was defined as any new postoperative motor or sensory deficit attributed to the surgical procedure, diagnosed on clinical examination by the treating surgeon at a routine postoperative visit within 90 days. Diagnosis was clinical throughout: confirmatory electrodiagnostic testing (electromyography or nerve conduction studies) was not required for case ascertainment and was not performed for the purposes of this study, and no structured screening instrument or standardized sensory mapping was administered. Because follow-up was limited to 90 days, cases could not be classified as transient or persistent; such symptoms after the DAA commonly resolve over a period extending beyond this interval, so the figures reported here describe incidence at 90 days and should not be read as rates of permanent deficit. Because ascertainment depended on symptoms being elicited and documented at a clinical encounter, and because sensory findings are subject to observer variability, the reported incidence should be regarded as a minimum estimate and is likely to underestimate true incidence relative to series using prospective structured screening.

Wound complications were defined as any wound problem identified within 90 days of the index procedure that required clinical treatment, rather than by descriptive label alone. Qualifying treatment comprised specific wound care (altered dressing regimens, in-office suture management, or negative-pressure therapy), oral or intravenous antibiotics, or operative management including revision closure or debridement. Presentations meeting this threshold included dehiscence, cellulitis, delayed incisional healing, and superficial surgical site infection. Wound findings that resolved without specific treatment were not counted. Deep periprosthetic infection was recorded separately and is not included in this category. Two groups of findings were excluded a priori: reactions attributable to the dressing rather than the wound, such as adhesive hypersensitivity, blistering, and contact dermatitis; and non-diagnostic observations recorded with uncertainty, such as queried small seromas noted in passing without associated intervention.

### Data analyses

Continuous variables were compared using parametric or non-parametric tests as appropriate to their distribution, and categorical variables using the chi-square or Fisher’s exact test as appropriate to expected cell counts. Trends in operative time across sequential case quartiles were assessed with analysis of variance. Multivariable logistic regression was used to model the odds of sustaining at least one complication, and multivariable linear regression to model operative time, each adjusting for age, BMI, and ASA class. Underlying diagnosis was available from the institutional arthroplasty registry and is reported in Table 1, but was not entered as a covariate because the cohorts did not differ materially in diagnostic composition and non-osteoarthritic diagnoses were too infrequent to model. A priori power analysis concluded that a minimum of 269 patients per group would be sufficient to detect a 5% absolute difference in overall complication rates given an alpha of 0.05 and 80% power [19]. This calculation defines the study’s resolution: it was designed to exclude a large difference, not to demonstrate equivalence, and no equivalence or non-inferiority margin was prespecified. Statistical significance was defined as a two-sided *p* < 0.05.

**Table 1:** Demographic characteristics by surgeon group

Variable	Junior Surgeons (*n* = 300)	Senior Surgeon (*n* = 300)	*p*-value
Age, years	63.3 ± 13.10	67.6 ± 9.49	< 0.001
Female, n (%)	157 (52.3%)	170 (56.7%)	0.325
BMI, kg/m^2^	27.9 ± 5.32	27.9 ± 5.24	0.985
ASA class	2.3 ± 0.68	2.4 ± 0.54	0.350
Primary osteoarthritis, n (%)	277 (92.3%)	273 (91.0%)	0.658
Osteonecrosis, n (%)	14 (4.7%)	17 (5.7%)	0.713
Post-traumatic, n (%)	7 (2.3%)	4 (1.3%)	0.545
Developmental dysplasia, n (%)	0 (0.0%)	3 (1.0%)	0.249
Inflammatory arthritis, n (%)	0 (0.0%)	1 (0.3%)	1.000

Values are mean ± SD. *p*-values from Welch’s independent samples t-test. Underlying diagnosis was abstracted from ICD-10 codes in the institutional arthroplasty registry and was recorded for all 600 patients. Sex was compared using the chi-square test and diagnosis categories using Fisher’s exact test

### Ethical approval

This study received institutional review board approval: IRB-AAAV1356.

## Results

### Patient demographics

A total of 600 patients were included in this study: 300 cases performed by three junior fellowship-trained surgeons and 300 cases performed by one fellowship-trained senior surgeon. Demographic characteristics are summarized in Table 1. The cohorts were well matched for BMI (27.9 ± 5.32 vs 27.9 ± 5.24 kg/m^2^, *p* = 0.985) and ASA classification (2.3 ± 0.68 vs 2.4 ± 0.54, *p* = 0.350). A statistically significant difference in mean age was identified, with junior cohort patients being younger than senior cohort patients (63.3 ± 13.10 vs 67.6 ± 9.49 years, respectively; *p* < 0.001). Age was adjusted for in all multivariable analyses; because comorbidity burden and preoperative hemoglobin were not available for adjustment, residual confounding by unmeasured case complexity cannot be excluded and is addressed in the Limitations. The cohorts were similar in underlying diagnosis: primary osteoarthritis accounted for 92.3% of junior and 91.0% of senior cases, with no significant difference in any diagnostic category (Table 1). All three dysplastic hips and the single inflammatory arthritis case were in the senior cohort, so the more complex diagnoses were not concentrated among the junior surgeons. Cemented femoral fixation was used in 80 of 600 cases (13.3%), including hybrid constructs, and was more common in the Junior cohort than the Senior cohort (15.0% vs 11.7%), although the difference was not statistically significant (*p* = 0.280).

### Operative complications

Operative complications at 90-day follow-up are presented in Table 2. Operative complication events were infrequent in both cohorts. There were 27 events among Junior cases (9.0 per 100 procedures) and 20 among Senior cases (6.7 per 100 procedures); the difference was not statistically significant (absolute difference + 2.3%, 95% CI − 2.0% to + 6.6%, *p* = 0.362). Because the study was powered to detect a 5% absolute difference, this result indicates no evidence of a large between-group difference; it does not establish equivalence, and the confidence interval remains compatible with a clinically relevant increase of up to approximately 7 percentage points in the Junior cohort. Dislocation was observed in 4 junior patients (1.3%) and 1 senior patient (0.3%, *p* = 0.373). Periprosthetic fracture occurred in 5 junior (1.7%) and 3 senior patients (1.0%, *p* = 0.725). Neurapraxia was identified in 4 junior (1.3%) and 6 senior patients (2.0%, *p* = 0.752). Prosthetic joint infection occurred in 3 junior patients (1.0%) and 1 senior patient (0.3%, *p* = 0.624). Wound complications occurred in 10 junior patients (3.3%) and 6 senior patients (2.0%, *p* = 0.448). One mortality occurred in each cohort (0.3% vs 0.3%, *p* = 1.000). Unplanned return to the operating room was required in 6 junior patients (2.0%) and 7 senior patients (2.3%, *p* = 1.000). Fisher’s Exact Test was used for all categorical comparisons given low expected cell frequencies.

**Table 2:** Operative complications at 90-day follow-up

Complication	Junior *n* (%)	Senior *n* (%)	*p*-value
Hematoma	1 (0.3%)	3 (1.0%)	0.624
Dislocation	4 (1.3%)	1 (0.3%)	0.373
Periprosthetic fracture	5 (1.7%)	3 (1.0%)	0.725
Neurapraxia	4 (1.3%)	6 (2.0%)	0.752
Prosthetic joint infection	3 (1.0%)	1 (0.3%)	0.624
Wound complication	10 (3.3%)	6 (2.0%)	0.448
Total operative complications	27 (9.0%)	20 (6.7%)	0.362
Patients with > = 1 operative complication	24 (8.0%)	18 (6.0%)	0.424
Return to OR	6 (2.0%)	7 (2.3%)	1.000

Fisher’s Exact Test throughout. OR = operating room. Counts are complication events; a patient sustaining more than one complication contributes to each applicable row. The composite comprises the six categories above it and totals 27 events (Junior) and 20 (Senior). Return to the operating room is shown for completeness but is excluded from the composite, because it is a consequence of the complications listed rather than an independent event and would double-count the patients concerned. Mortality is reported with the medical complications in Table 3. The final row gives the number of patients with at least one operative complication, which is lower than the event total because some patients sustained more than one

### Medical complications

Medical complications at 90-day follow-up are presented in Table 3. No statistically significant differences were identified between groups for any individual medical complication. Pulmonary embolism occurred in 1 junior patient (0.3%) and 1 senior patient (0.3%, *p* = 1.000). Deep vein thrombosis occurred in 3 junior patients (1.0%) versus 2 senior patients (0.7%, *p* = 1.000). Transfusion was required in 5 junior patients (1.7%) versus 3 senior patients (1.0%, *p* = 0.725). Stroke occurred in 2 senior patients (0.7%) and no junior patients (*p* = 0.499). Acute kidney injury was observed in 2 junior patients (0.7%) and 3 senior patients (1.0%, *p* = 1.000). Urinary retention occurred in 1 junior patient (0.3%) and 3 senior patients (1.0%, *p* = 0.624). Myocardial infarction and pneumonia were each absent in the junior cohort, and each occurred in 1 senior patient (*p* = 1.000 for both). In aggregate, 13 medical events were recorded in the junior cohort (4.3 per 100 procedures) and 17 in the senior cohort (5.7 per 100 procedures, *p* = 0.575); these figures include the single death in each cohort. Combining operative and medical events, 40 complication events occurred among the 300 junior THAs (13.3 per 100 procedures) and 37 among the 300 senior THAs (12.3 per 100 procedures; absolute difference + 1.0%, 95% CI − 4.4% to + 6.4%, *p* = 0.807). Return to the operating room occurred in 6 junior (2.0%) and 7 senior (2.3%) cases (*p* = 1.000) and is excluded from these totals for the reason given above. The figures reported are counts of events, not counts of patients with at least one complication; because several patients sustained more than one complication, the patient-level proportion is necessarily lower and is reported separately in Table 3. The similar composite event counts in the two cohorts should not be read as evidence of equivalence: with 300 patients per group, the confidence interval around this difference spans approximately five percentage points in either direction, so the data remain compatible with a clinically relevant difference that this study could not detect.

**Table 3:** Medical complications and overall complication rate at 90-day follow-up

Complication	Junior *n* (%)	Senior *n* (%)	*p*-value
Deep vein thrombosis	3 (1.0%)	2 (0.7%)	1.000
Pulmonary embolism	1 (0.3%)	1 (0.3%)	1.000
Anemia requiring transfusion	5 (1.7%)	3 (1.0%)	0.725
Myocardial infarction	0 (0.0%)	1 (0.3%)	1.000
Stroke	0 (0.0%)	2 (0.7%)	0.499
Pneumonia	0 (0.0%)	1 (0.3%)	1.000
Urinary retention	1 (0.3%)	3 (1.0%)	0.624
Acute kidney injury	2 (0.7%)	3 (1.0%)	1.000
Mortality	1 (0.3%)	1 (0.3%)	1.000
Total medical complications	13 (4.3%)	17 (5.7%)	0.575
Any complication	40 (13.3%)	37 (12.3%)	0.807
Patients with > = 1 complication	33 (11.0%)	28 (9.3%)	0.589

Fisher’s Exact Test. Mortality is included among the medical complications. “Any complication” reflects the total number of complication EVENTS of any type, operative or medical (27 + 13 = 40 Junior; 20 + 17 = 37 Senior), and excludes return to the operating room, which is reported separately in Table 2. It is not the number of patients with at least one complication, which is given in the final row and is the outcome modelled in Table 4

### Multivariate analysis—complication risk

To evaluate the independent effect of surgeon experience on 90-day complication risk while accounting for the observed age difference between cohorts, logistic regression was performed adjusting for age, BMI, and ASA classification Table 4. Surgeon group was not a significant independent predictor of sustaining at least one complication at 90 days (OR 1.43, 95% CI 0.84–2.46, *p* = 0.190). Age and BMI were likewise not significant independent predictors (*p* = 0.244 and *p* = 0.473). Higher ASA class was associated with increased complication risk, at the margin of conventional significance (OR 1.59 per class, 95% CI 1.00–2.55, *p* = 0.051). The direction of the surgeon-group estimate is consistent with the unadjusted comparison, but its confidence interval is wide and includes values in both directions; with 61 patients sustaining a complication across the whole cohort, this model is not well powered to isolate a surgeon-group effect.

**Table 4:** Logistic regression—any complication at 90 days (*n* = 600)

Variable	OR	95% CI	*p*-value
Surgeon group (Junior vs Senior)	1.43	0.84–2.46	0.190
Age (per year)	1.01	0.99–1.04	0.244
BMI (per unit)	1.02	0.97–1.07	0.473
ASA class (per point)	1.59	1.00–2.55	0.051

Reference group: senior surgeon. OR = odds ratio; CI = confidence interval. Pseudo-R2 (McFadden) = 0.020. Outcome is the presence of at least one complication at 90 days (patient level). Adjustment was limited to age, BMI, and ASA class; comorbidity index, preoperative hemoglobin, femoral morphology, and bone defects were not available in the retrospective dataset; underlying diagnosis was available and did not differ between cohorts, and was therefore not modelled. The wide confidence interval reflects limited power and is compatible with a clinically relevant difference in either direction. The model was fitted on all 600 patients

### Operative time

Operative time data are presented in Table 5. Mean operative time for the junior cohort combined was 92.3 ± 24.5 min (median 88 min, range 56–237) compared with 68.5 ± 16.2 min for the senior surgeon (median 68 min, range 36–141; *p* < 0.001, Mann–Whitney U), a mean difference of 23.8 min.

**Table 5:** Operative time by surgeon

Surgeon	Mean ± SD, min	Median	Range, min	*n*
Junior Surgeon 1	97.3 ± 27.6	92	56–237	100
Junior Surgeon 2	93.0 ± 24.9	90	56–188	100
Junior Surgeon 3	86.7 ± 19.5	86	56–162	100
Junior Combined	92.3 ± 24.5	88	56–237	300
Senior Surgeon	68.5 ± 16.2	68	36–141	300

Junior combined vs Senior: *p* < 0.001, Mann–Whitney U

Sequential quartile analysis demonstrated a significant efficiency learning curve across all junior surgeons Table 6. Combined junior Q1 mean operative time was 108.3 ± 25.1 min, declining to 84.6 ± 19.2 min in Q4 (ANOVA *F* = 17.35, *p* < 0.001; Q1 vs Q4, *p* < 0.001). There was no statistically significant difference in operative time between the three junior surgeons (*p* = 0.11, Kruskal–Wallis). In contrast, the senior surgeon’s operative time was stable across all four equal 75-case blocks (68.6, 72.0, 65.9, and 67.5 min, delta =  − 1.1 min), with no significant trend, consistent with an established operative plateau.

**Table 6:** Operative time learning curve by quartile (mean ± SD, minutes)

Surgeon	Q1 (Cases 1–25 (Junior) & Cases 1–75 (Senior)), min	Q2 (Cases 26–50 (Junior) & Cases 76–150 (Senior)), min	Q3 (Cases 51–75 (Junior) & Cases 151–225 (Senior))	Q4 (Cases 76–100 (Junior) & Cases 226–300 (Senior))
Junior Surgeon 1	120.0 ± 24.9	88.6 ± 16.9	96.0 ± 34.3	84.5 ± 16.2
Junior Surgeon 2	108.0 ± 28.1	86.4 ± 17.5	89.8 ± 23.9	87.6 ± 23.9
Junior Surgeon 3	97.0 ± 15.8	83.1 ± 16.8	85.2 ± 24.5	81.7 ± 17.0
Junior Combined	108.3 ± 25.1	86.0 ± 17.0	90.3 ± 28.0	84.6 ± 19.2
Senior Surgeon	68.6 ± 16.8	72.0 ± 18.0	65.9 ± 15.2	67.5 ± 14.2

Quartile analysis uses sequential case order per surgeon

### Multivariate analysis—operative time

Linear regression modelling of operative time is presented in Table 7. After adjusting for age, BMI, and ASA classification, junior surgeons performed cases a mean of 24.5 min longer than the senior surgeon (95% CI + 21.1 to + 27.8, *p* < 0.001). Higher BMI was independently associated with longer operative time, with each unit increase adding 0.6 min (95% CI + 0.3 to + 0.9, *p* < 0.001), consistent with increased technical complexity in higher body habitus patients undergoing the direct anterior approach. Age and ASA class were not significant independent predictors of operative time (*p* = 0.108 and *p* = 0.101, respectively).

**Table 7:** Linear regression—operative time in minutes (*n* = 600)

Variable	β, min	95% CI	*p*-value
Surgeon group (Junior vs Senior)	+ 24.5	+ 21.1 to + 27.8	< 0.001
Age (per year)	+ 0.1	−0.0 to + 0.3	0.108
BMI (per unit)	+ 0.6	+ 0.3 to + 0.9	< 0.001
ASA class (per point)	+ 2.3	−0.5 to + 5.1	0.101

Reference group: senior surgeon. β = unstandardized regression coefficient. R^2^ = 0.277, adjusted R^2^ = 0.272

### Patient-reported outcomes

At baseline, patients treated by junior surgeons had significantly worse SF-12 physical and WOMAC scores than those treated by the senior surgeon, whereas SF-12 mental scores were similar between cohorts. Preoperatively, SF-12 physical scores averaged 37.4 in the junior cohort and 41.1 in the senior cohort (*p* < 0.001). WOMAC pain, stiffness, and function scores were also lower (worse) in the junior cohort (38.0, 39.7, and 37.0, respectively) compared with the senior cohort (49.7, 51.6, and 50.2; all *p* < 0.001). In contrast, SF-12 mental scores were nearly identical at baseline (47.1 vs 46.9 for junior vs senior cohorts, *p* = 0.82) (Table 8).

**Table 8:** EXPLORATORY analysis of patient-reported outcome measures pre-operatively and at 90-day follow-up, including SF-12 and WOMAC metrics. Ninety-day follow-up was 35% (Junior) and 48% (Senior); paired change scores were available for 102 (34%) Junior and 141–143 (47–48%) Senior patients. Between-group comparisons of change scores are susceptible to differential non-response and to floor effects arising from lower baseline scores in the Junior cohort, and should not be interpreted as evidence of superior recovery

PROM	Time point	Junior (mean ± SD, *n*)	Senior (mean ± SD, *n*)	*p*-value (Junior vs Senior)
SF-12 Physical	Pre-op	37.35 ± 7.90 (*n* = 262)	41.15 ± 7.49 (*n* = 284)	< 0.001
	3-month	46.24 ± 7.63 (*n* = 105)	46.70 ± 7.32 (*n* = 144)	0.630
	Change (3-month—pre-op)	8.59 ± 8.92 (*n* = 102)	6.20 ± 7.50 (*n* = 143)	0.029
SF-12 Mental	Pre-op	47.06 ± 6.20 (*n* = 262)	46.93 ± 7.19 (*n* = 284)	0.822
	3-month	47.62 ± 5.70 (*n* = 105)	46.51 ± 6.97 (*n* = 144)	0.169
	Change (3-month—pre-op)	0.11 ± 8.12 (*n* = 102)	−0.48 ± 8.54 (*n* = 143)	0.584
WOMAC Pain	Pre-op	38.02 ± 21.55 (*n* = 260)	49.74 ± 20.67 (*n* = 278)	< 0.001
	3-month	87.43 ± 16.23 (*n* = 105)	89.15 ± 15.10 (*n* = 143)	0.396
	Change (3-month—pre-op)	50.15 ± 24.28 (*n* = 102)	38.12 ± 23.20 (*n* = 141)	< 0.001
WOMAC Stiffness	Pre-op	39.73 ± 24.05 (*n* = 259)	51.64 ± 28.10 (*n* = 279)	< 0.001
	3-month	75.95 ± 20.78 (*n* = 105)	78.80 ± 21.63 (*n* = 146)	0.293
	Change (3-month—pre-op)	36.27 ± 31.93 (*n* = 102)	28.86 ± 31.89 (*n* = 142)	0.075
WOMAC Function	Pre-op	36.96 ± 19.18 (*n* = 258)	50.23 ± 23.59 (*n* = 278)	< 0.001
	3-month	81.78 ± 17.08 (*n* = 105)	84.14 ± 19.31 (*n* = 146)	0.307
	Change (3-month—pre-op)	44.40 ± 23.61 (*n* = 102)	32.91 ± 26.35 (*n* = 141)	< 0.001

By 90 days, both cohorts demonstrated large improvements in pain and function, and cross-sectional PROMs were similar between junior and senior surgeons. SF-12 physical scores at 90 days were 46.2 in the junior cohort and 46.7 in the senior cohort (*p* = 0.63), and SF-12 mental scores were 47.6 versus 46.5 (*p* = 0.17). WOMAC pain, stiffness, and function at 90 days were likewise comparable between groups (pain 87.4 vs 89.2, *p* = 0.40; stiffness 76.0 vs 78.8, *p* = 0.29; function 81.8 vs 84.1, *p* = 0.31 for junior vs senior cohorts, respectively) (Table 8).

In the subset of patients with paired preoperative and 90-day follow-up data, absolute improvements in several PROMs were greater in the junior cohort. Among 102 junior and 143 senior patients with paired SF-12 physical scores, SF-12 physical increased by 8.6 points in the junior cohort compared with 6.2 points in the senior cohort (*p* = 0.029). Improvements in WOMAC pain and function were also significantly larger for junior surgeons, with WOMAC pain increasing by 50.1 vs 38.1 points (*n* = 102 and 141, respectively; *p* < 0.001) and WOMAC function increasing by 44.4 vs 32.9 points (*p* < 0.001). Differences in absolute change for SF-12 mental (0.1 vs − 0.5 points; *p* = 0.58) and WOMAC stiffness (36.3 vs 28.9 points; *p* = 0.07) were not statistically significant. Because these subsets differ in size and in baseline severity between cohorts, the between-group comparison of change scores is interpreted in the Discussion rather than here. Paired change scores were available for 102 (34%) junior and 141–143 (47–48%) senior patients Table 8.

All patient-reported outcome analyses in this section are exploratory. Ninety-day PROM follow-up was 35% in the junior cohort and 48% in the senior cohort, and paired pre-/post-operative data were available for only 102 junior (34%) and 141–143 senior (47–48%) patients. With loss to follow-up of this magnitude, non-response bias cannot be excluded. These analyses are therefore reported to describe the observed subset, and are not a basis for inference about between-group differences in functional recovery.

## Discussion

Historically, the DAA has been characterized by increased complications during early adoption, with outcomes improving over the first 50–100 procedures, particularly among surgeons who learned the approach after fellowship [7, 8]. In our cohort, junior surgeons had significantly longer operative times but no detected difference in 90-day complication rates. These findings are compatible with, but do not demonstrate, attenuation of the historical safety learning curve with contemporary fellowship training. The observed 2.3 percentage-point difference reflected a numerically higher rate in the Junior group, but did not reach statistical significance. Because the study was powered only to detect a 5% absolute difference, a non-significant result does not establish equivalence.

Prior series have described early clustering of femoral fractures, lateral femoral cutaneous nerve neurapraxia, component malposition, and wound complications during the first 50–100 cases [7–18]. We did not detect a significant difference between junior and senior surgeons despite longer operative times in the junior cohort (9.0% vs 6.7%; *p* = 0.362). At the observed complication rate, 300 cases per group provided 80% power only for a difference of approximately 6.8 percentage points, leaving limited power for smaller differences and rare events. These data therefore provide no evidence of a large safety penalty in this setting but cannot exclude a smaller clinically important difference. Our rates also should not be directly compared with the 2.75% pooled complication prevalence reported by Corradi et al. across 96,605 DAA THAs, because that review was dominated by major events whereas our composite included all documented 90-day adverse events, including superficial wound complications, which accounted for 10 of 27 junior events [20]. The review identified heterogeneity in complication definitions as a major barrier to comparison and reported an average threshold of 86 cases at which complication rates and operative time declined, placing our 100-case junior series within the conventional interval over which the early learning curve is considered to resolve [20].

Several features of the contemporary surgical environment may contribute to these findings. Burnham et al. studied 286 DAA THAs performed by four fellowship-trained surgeons during their first independent year and found that fellowship training eliminated learning effects for operative time and blood loss, although a complication-based learning curve persisted, with surgical complications declining after approximately 40 cases and medical complications normalizing by 25–50 cases [21]. We did not detect a corresponding complication difference, but our junior-versus-senior design did not test case-order effects within the junior cohort and was not powered to detect differences of the magnitude they described. Whether continued evolution in fellowship training intensity and institutional perioperative infrastructure further compresses the safety curve remains uncertain. Our findings therefore complement rather than contradict prior work: they address whether the first 100 independent cases of fellowship-trained surgeons differ from an experienced comparator, not whether complications decline within those 100 cases. The distinction between an efficiency and safety curve is therefore central to interpreting these findings: fellowship training may reduce major early complications while operative efficiency continues to improve during the first 100 independent cases. Our data raise this possibility but cannot determine whether it is attributable to training, institutional systems, or individual surgeon factors.

Garbarino et al. prospectively followed the first 200 cases of a fellowship-trained surgeon and found differences in operative time, component positioning, and neurapraxia between the first and second 100 cases [22]. Their comparison with historical cohorts suggested that fellowship training reduced early fracture and reoperation rates. Their neurapraxia rate fell from 41% to 9%, a signal not replicated here. Ascertainment is an important explanation: our cases were identified from symptoms documented at routine clinical encounters without structured sensory screening, making our rates minimum estimates and not directly comparable with prospectively screened series. Technical refinement during fellowship may contribute, but cannot be separated from ascertainment in our data. This is particularly relevant because their prospectively screened neurapraxia rate was substantially higher than ours, underscoring how outcome ascertainment can influence apparent learning-curve magnitude. Our broader clinical definition captured motor or sensory deficits attributable to surgery, but without structured screening or electrodiagnostic testing, the present estimate likely represents a minimum incidence.

Messina et al. compared the first 100 cases of six fellowship-trained surgeons using the DAA and posterolateral approaches and found no significant differences in revision, surgical complication, or total complication rates, although both approaches had higher complication rates during their first 50 cases [19]. Our study likewise did not identify an early safety signal, but did not formally test case-order effects. One possible explanation is that institutional factors beyond fellowship training, including standardized ERAS pathways and high-volume center experience, may have additive effects on early outcomes. Our single-center design cannot separate these factors from training effects, and this hypothesis requires testing across centers with differing perioperative infrastructure. The absence of a within-surgeon case-order analysis also means that a safety curve occurring early within the Junior series could have been missed by the between-group comparison.

ERAS and standardized perioperative protocols may reduce variability and common early pitfalls, potentially narrowing differences between surgeons with different levels of experience [23–25]. ERAS recommendations and subsequent reviews in arthroplasty associate protocolized care with shorter stays and reductions in complications and cost [23]. In a setting where fellowship training and ERAS infrastructure co-evolve, operative time may remain the most apparent early differentiator, while safety differences become more difficult to detect.

The exploratory PROM findings should be interpreted cautiously because 90-day follow-up was below 50% in both cohorts. Junior patients had worse baseline pain, stiffness, function, and SF-12 physical scores, while 90-day scores among respondents were comparable. The larger absolute changes observed in several junior PROMs should not be interpreted as superior recovery. Paired data were available for only about one third of junior and one half of senior patients, and differential questionnaire response could favor patients with greater improvement. Because the junior cohort began with worse baseline scores, this mechanism could selectively inflate observed junior change scores. In addition, lower baseline scores mathematically permit larger absolute change; junior baseline WOMAC pain was 11.7 points worse, so a floor effect alone would predict part of the observed difference. Poor recovery or complications may also reduce questionnaire completion, enriching the responding subset for better outcomes in either cohort. Although available demographic data for PROM completers and non-completers did not differ significantly in age, BMI, or ASA, preoperative functional status could not be compared, and response bias related to outcome cannot be excluded. The 90-day absolute scores are less vulnerable to baseline change-score effects, but they remain subject to non-response bias. With this degree of attrition and baseline imbalance, these analyses cannot support conclusions about functional recovery. Prospective studies with higher capture rates and designs controlling patient-level factors, such as bilateral staged THA with each patient serving as their own control [6], are better suited to this question. The cross-sectional 90-day scores are therefore more appropriately viewed as descriptive of respondents, while the change-score findings should be considered particularly vulnerable to baseline and response differences.

This study has important limitations. First, the Senior cohort represents one surgeon whereas the Junior cohort represents three, so any seniority effect is inseparable from individual surgeon performance, case selection, and practice patterns and may not generalize to experienced DAA surgeons. Second, adjustment was limited to age, BMI, and ASA class. Although underlying diagnosis did not differ between cohorts, Deyo-Charlson comorbidity index, preoperative hemoglobin, prior ipsilateral hip surgery, Crowe grading of dysplastic hips, Dorr femoral morphology, and acetabular or femoral bone defects were unavailable. Residual confounding by comorbidity and anatomic complexity therefore cannot be excluded, particularly because the Junior cohort was significantly younger. Third, implant design and fixation were not uniform and were confounded by surgeon. Cemented fixation was somewhat more common in the Junior cohort (15.0% vs 11.7%, *p* = 0.280), which could favor the Junior cohort for periprosthetic fracture, yet Junior surgeons had more fractures (5 vs 3). Cemented fixation also reflects bone quality and may therefore mark unmeasured case complexity. Fracture-related differences cannot be attributed to surgeon experience alone. Fourth, 90-day follow-up may miss later complications such as late dislocation, aseptic loosening, or revision, and the single high-volume center with established ERAS pathways may limit generalizability. The cohort’s relatively low BMI (27.9 kg/m^2^ in both groups) compared with national THA populations may also limit applicability to higher-BMI patients, for whom DAA exposure and soft-tissue management may be more challenging. PROMs were available only for a subset; completers and non-completers did not differ in age, BMI, or ASA, but preoperative functional status could not be compared, so response bias remains possible. Radiographic measures of component position, offset, and leg length were not analyzed and remain important future endpoints.

Finally, although the sample exceeded the a priori target, the observed complication rate meant that 300 cases per group provided 80% power only for a 6.8-point difference; individual rare events and smaller differences remain underpowered. These limitations reinforce that the study is best interpreted as hypothesis-generating and specific to a high-volume center with standardized perioperative care.

## Conclusion

In this single-center retrospective cohort, three fellowship-trained surgeons performing their first 100 DAA THAs had operative times approximately 24 min longer than one experienced high-volume DAA surgeon, with no evidence of a large difference in 90-day complication rates. Because the study was powered only to detect a 5% absolute difference in overall complications, it cannot exclude smaller differences or differences in individually rare events, and it does not establish equivalence. Exploratory patient-reported outcome analyses, limited by 90-day follow-up below 50% in both cohorts, are hypothesis-generating only and do not support conclusions about functional recovery. What these data show is narrower than the question they were designed to address: within a single high-volume center operating under standardized ERAS pathways, three fellowship-trained surgeons early in independent practice had complication rates that were not significantly different from those of one experienced surgeon, with the caveat that the study cannot exclude smaller or rare-event differences. These observations nonetheless generate a specific and testable hypothesis—that in this setting the contemporary DAA learning curve is principally one of operative efficiency rather than safety, and that training quality and institutional infrastructure may be more important determinants of early outcome than surgeon seniority alone. Adequately powered multicenter studies, with complete follow-up and adjustment for comorbidity and anatomical complexity, are required before this hypothesis can be regarded as established or used to inform training or credentialing policy.

## Data Availability

No datasets were generated or analysed during the current study.
